# Fluorescence-Guided Chitosan and Eugenol-Based Carbon Dots for Comprehensive Infection Control and In Vitro Wound-Healing Applications in Dentistry

**DOI:** 10.3390/dj14030133

**Published:** 2026-02-26

**Authors:** Navya Narayanan, Aruchamy Mohanprasanth, Natesan Thirumalaivasan

**Affiliations:** Applied Nano-Bio Therapeutics Laboratory, Department of Periodontics, Saveetha Dental College and Hospitals, Saveetha Institute of Medical and Technical Sciences (SIMATS), Saveetha University, Chennai 600077, Tamil Nadu, India; navyanarayanan146@gmail.com (N.N.); mohanbiochemist03@gmail.com (A.M.)

**Keywords:** *Streptococcus mutans*, carbon dots, chitosan, eugenol, antimicrobial activity and wound healing

## Abstract

**Background/Objectives:** The transformation of multifunctioning nanomaterials, incorporating antimicrobial activity and regenerative incompatibility, is becoming even more significant in the modern dental therapeutic context. *Streptococcus mutans* (*S. mutans*) is primarily linked to dental caries and pulp inflammation and requires new strategies that would be efficient at controlling the infection and promoting tissue repair. The objectives of the present research were to synthesize and critique the use of chitosan–eugenol carbon dots (CECDs) as a versatile nanoplatform in the field of dentistry to implement antimicrobial and regenerative dentistry. **Methods:** CECDs synthesized from biocompatible chitosan and eugenol were characterized by Scanning Electron Microscopy (SEM) and Energy Dispersive X-ray (EDX), evaluated from *S. mutans* inhibition via MIC/MBC, and assessed from cytocompatibility using 3T3-L1 fibroblast viability, morphology, and migration assays. **Results:** The resultant CECDs had a spherical morphology and a size of 5 ± 2 nm. The EDX analysis established the existence of carbon, nitrogen and oxygen labeling successful incorporation of heteroatoms, as well as surface functionalization. CECDs exhibited greater antibacterial effects against *S. mutans* through a concentration-dependent approach with MIC and MBC of 125 and 250 µg/mL respectively. Cytotoxicity assays indicated that the cells were viable, their morphology was intact, and that the cells moved more vigorously, which confirmed excellent biocompatibility. **Conclusions:** The synergistic combination of chitosan and eugenol into the carbon dot structure produced CECDs that had strong biomarker along with antibacterial activity and desirable cytocompatibility. These results indicate that CECDs are an attractive multifunctional nanoplatform in the treatment of oral infections and help with wound healing.

## 1. Introduction

Oral infections are one of the greatest worldwide public health burdens and have immense impacts on both oral and systemic health. The oral cavity is a highly dynamic microbial state that harbors diverse communities of bacteria, fungi, viruses, and protozoa that live in a finely regulated ecological balance that is essential for the maintenance of oral homeostasis [1]. Perturbation of this equilibrium (resulting from inadequate oral hygiene, immunosuppression, metabolic/systemic diseases, dietary influences or environmental stressors) can favor dysbiosis and overgrowth of pathogen microorganisms, which results in prevalent oral diseases such as dental caries, gingivitis, periodontitis or odontogenic abscesses [2]. Among these, dental caries and periodontal diseases are the most diffuse and clinically relevant with a consequential contribution to chronic pain, tooth loss, dysfunction of mastication—especially limited—and a marked reduction in quality of life. Dental caries is a biofilm mediated multifactorial disease, which is characterized by progressive demineralization of the enamel due to organic acids produced by the fermentation of dietary carbohydrates by acidogenic and aciduric bacteria (predominantly *Streptococcus Mutans* and *Lactobacillus* species). When left untreated, caries can lead to pulpitis, periapical abscess, and systemic manifestations such as transient or persistent bacteremia; thus, there is a need for effective strategies of prevention and treatment.

Current clinical management of oral infections is based on integrated preventive, antimicrobial and regenerative strategies, such as mechanical plaque clearance, professional dental prophylaxis, anti-septic mouth rinses and systemic antibiotics or localized antibiotic treatment [3]. But the growing problem of antibiotic-resistant strains of microbes is a critical threat to the long-term effectiveness of traditional treatment methods, and there is a need to develop alternative and sustainable antimicrobial interventions. In response, innovative approaches are actively explored in order to act as natural bioactive compounds, probiotics, photodynamic therapy (PDT), and nanotechnology-based antimicrobial platforms. Nanomaterials in particular provide the potential to simultaneously add antimicrobial activity, biofilm disruption, targeted drug delivery and tissue regenerative support—features of particular relevance in tackling the complex pathophysiology of oral infections.

Among emerging nanomaterials, carbon dots (CDs) have attracted considerable attention as a versatile class of carbon-based nanoparticles with unique physicochemical, optical and biological properties. CDs are quasi-spherical nanoparticles that are typically less than 10 nm in size with a composition of carbon and oxygen plus optionally heteroatom doping (e.g., nitrogen, sulfur or phosphorus depending on synthesis route, e.g., [4,5]). They possess good and tunable photoluminescence, excitation-dependent fluorescence, high aqueous dispersibility, chemical stability, and high biocompatibility as promising candidates for bioimaging, biosensing, drug delivery, and antimicrobial application. Compared with conventional semiconductor quantum dots, the cytotoxicity of CDs is significantly lower, there is higher environmental safety, and better functional tunability to adapt to clinical translation [6]. Importantly, through size control, surface passivation, and heteroatom incorporation they can be precisely engineered to possess all aspects of optical properties which enables multi-color emission and responsive imaging under a single excitation source. Their high surface functional group density (OH, COOH, NH_2_) favors effective conjugation with therapeutic agents and biomolecules and their tunable surface charge allows the interaction to be increased with microbial membranes and host cells. Notably, the CDs are capable of generating reactive oxygen species (ROS) under light activation and thus have intrinsic antimicrobial and antibiofilm activity [7]. Their nanoscale dimensions and high surface area to volume ratio further facilitate good bacterial membrane disruption and uptake into cells and thereby are of much interest for dental applications, where high antimicrobial performance and regenerative potentials are key [8].

**Figure 1:**
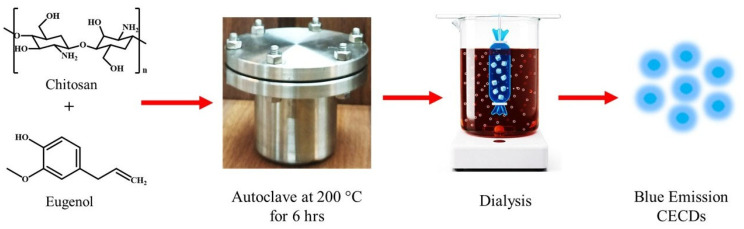
Schematic illustration of CECDs preparation.

At the same time, phytochemicals of plant origin, including eugenol, which is a constituent of clove (*Syzygium aromaticum*) oil, have shown effective antimicrobial, antioxidant, anti-inflammatory, and analgesic effects and have long been used in dental formulations, including dentifrices, restorative cements, oral rinses, and even temporary filling materials [9]. The antimicrobial acts of eugenol include membrane alteration and oxidative-stress-pathway manipulation and the anti-inflammatory mechanism is counteracted by wound healing, analgesia, and cytokine regulation. Despite its therapeutic promise, a clinical use of free eugenol is constrained due to its high volatility, low aqueous solubility, rapid degradation and low biological half-life [10]. In order to address these shortcomings, the current study will focus on creating a new nanocarrier-based delivery system that ensures the stability of eugenol and allows its controlled delivery by incorporating a biocompatible polymer like chitosan or carbon dot nanostructures (Figure 1). The synergistic strategy is aimed to prevent fomenting antimicrobial effects, enhance antibiofilm activity and foster tissue regeneration, thus providing a next-generation strategy of the dental and regenerative medicine management of oral infections.

## 2. Material and Methods

### 2.1. Materials

Chitosan (high molecular weight, degree of deacetylation ≥ 75%, Cat. No: 419419) was purchased from Sigma-Aldrich (St. Louis, MO, USA). Eugenol (purity ≥ 99%, Lot No: A0232) was procured from Tokyo Chemical Industry (TCI) (Tokyo, Japan). Mueller–Hinton agar (Cat. No: M173), and Mueller–Hinton broth (Lot No: M391) were obtained from HiMedia Laboratories (Mumbai, India). Dulbecco’s Modified Eagle Medium (DMEM; Lot No: 289134) and fetal bovine serum (FBS; Lot No: 2774559) were purchased from Gibco, Thermo Fisher Scientific (Waltham, MA, USA). Penicillin–streptomycin solution (Lot No. A002-100 mL) was purchased from HiMedia Laboratories (Mumbai, India). All additional reagents and solvents used in the study were of analytical grade and sourced from authorized local vendors.

### 2.2. Methods

#### 2.2.1. Preparation of Chitosan and Eugenol-Based Carbon Dots

CECDs were prepared using a controlled hydrothermal carbonization approach that is aimed at facilitating efficient heteroatom doping and surface passivation. Briefly, chitosan 200 mg in 20 mL deionized water with the addition of eugenol (0.3 mL), a bioactive phenolic precursor, which allows the formation of conjugated C-N and C-O functional domains important in antimicrobial activity, was done. The mixture was sonicated for 10 min to provide homogenous mixing at the molecular level and stirred overnight to provide for the pre-polymerization and hydrogen-bond-mediated self-assembly. The resultant solution was placed in a Teflon-lined autoclave and underwent hydrothermal treatment at 200 °C for 6 h, which included a dehydration process as well as an aromatization and nucleation process resulting in the formation of fluorescent CDs. After natural cooling, the reaction product was centrifuged and filtered to remove large particulates, and then followed by dialysis (1 kDa cutoff) to remove any unreacted by-products and unreacted precursors, all of low molecular weight. The purified suspension was further centrifuged at 6000 rpm for 20 min and the obtained CECDs pellet was sequentially washed with ethanol and distilled water in order to remove surface-adsorbed impurities. The resulting CECDs were stored at ambient temperature, providing stable and highly dispersible photoluminescence applicable to the subsequent biomedical applications considering the usage of membrane disruption, reactive oxygen species generation and biofilm inhibiting pathways [11].

#### 2.2.2. Characterization of Synthesized CECDs

Physicochemical properties of carbon dots are essential factors in defining their efficacy, biodistribution and mechanism of action. Thus, adequate profile characterization of the CECDs was carried out to assess the functional characteristics of the compounds on the basis of different analysis methods. A UV-visible spectrophotometer (Thermo Scientific Evolution 600) with 1 cm quartz cuvettes was used to determine the optical properties of the synthesized Carbon dots, and absorbance spectra were recorded over 200–800 nm. Fourier Transform Infrared (FTIR) spectroscopy was used to measure the functional groups of CECDs at the 4000–400 cm^−1^ spectrum using a Bruker spectrophotometer. Moreover, X-ray diffraction was used to identify the crystalline nature and phase of the carbon dots and revealed distinct diffraction peaks from the crystal lattice of the phytochemical-mediated Carbon dots. Scanning Electron Microscopy (SEM) (JSM-7001F, JEOL, Tokyo, Japan) in an accelerating voltage of 20 keV was used to study the surface morphology of the synthesized Carbon dots. The elemental composition and purity were again confirmed by means of the Energy Dispersive X-ray (EDX) spectroscopy (JSM-7001F, JEOL, Tokyo, Japan), which confirmed that the sample contained elements that are linked to chitosan and eugenol. The fluorescence characteristics, such as excitation maximum and emission maximum, were investigated. Emission (photoluminescence) spectroscopy was used for measuring photostability ensured under continuous light exposure to that of the emission stability.

#### 2.2.3. Antimicrobial Activity Against Oral Pathogen *S. mutans*

##### Agar Well Diffusion Method

The agar well diffusion technique was utilized to establish the inhibitory effect of CECDs against oral pathogen *S. mutans*. The bacterial cultures were incubated in the Mueller–Hinton (MH) broth for 18 h of 37 °C and the turbidity was equilibrated with 0.5 McFarland standard. The Mueller–Hinton Agar (MHA) agar plates were prepared by pouring a medium of distilled water under 300 mL then sterilizing the medium through autoclave. After solidification, the bacterial suspension was swabbed evenly on the plates. Wells were prepared using sterile tips and loaded into the various concentrations of CECDs (20–80 µg/mL), and the positive control was Ciprofloxacin. Then, the plates were incubated at 37 °C for 24 h and the zones of inhibitions were documented to demonstrate the level of antibacterial activity of the test samples [12].

##### Minimum Inhibitory Concentration (MIC)

The Minimum Inhibitory Concentration (MIC) of CECDs was determined using a twofold serial dilution technique in a sterile 96-well microtiter plate. A stock solution of CECDs (1 mg/mL) was prepared in sterile deionized water. After that, 0.1 mL of Mueller–Hinton Broth (MHB) was transferred to each well and serial twofold dilutions were made to reach 1000–1.95 µg/mL. A bacterial culture in MHB was grown overnight at 37 °C, after which it was adjusted to 0.5 McFarland standard (~1.5 × 10^8^ CFU/mL) and diluted 1:100 in fresh broth to produce a final inoculum of about 1 × 10^6^ CFU/mL. Afterwards, 100 µL of bacterial suspension was added to each well, for a total volume of 200 µL per well. The positive control (bacteria in the absence of the test compound), negative control (medium in the presence of the test compound and without bacteria), and an antibiotic control (Ciprofloxacin, 10 µg/mL) were used. The plate was incubated at 37 °C for 18–24 h under aerobic conditions, and the wells were inspected for turbidity. The lowest concentration of CECDs that completely inhibited visible bacterial growth was determined as the MIC relative to the controls. To confirm this, 20 µL of resazurin dye (0.015% *w*/*v*) was added to each well and incubated for 2 h. The change in color from blue to pink showed the presence of bacteria and the change in color to another was a confirmation that all the microbial activities were inhibited [13].

##### Minimum Bactericidal Concentration (MBC)

The Minimum Bactericidal Concentration (MBC) was calculated by subculturing wells that had no visible bacterial growth in the MIC assay. A 10 µL of each well was aseptically transferred to Mueller–Hinton Agar (MHA) plates and incubated at 37 °C for 24 h. The minimum concentration of test compound that did not obtain a visible bacterial colony on the surface of the agar was taken as the MBC.

##### Biofilm Formation by Confocal Laser Scanning Microscopy (CLSM)

After the incubation period, the culture medium was carefully removed and the wells were gently washed with 1× PBS to eliminate non-adherent cells. The biofilms were then incubated with SYTO9 and propidium iodide staining solution for 15 min in the dark at room temperature. Excess stain was removed, samples were washed again with 1× PBS, and biofilm formation was subsequently observed using confocal laser scanning microscopy (CLSM).

#### 2.2.4. Cytotoxicity Assay

##### Cell Line

The mouse embryonic fibroblast (3T3-L1 cell line) was the one ordered from NCCS, Pune, India. The cells were grown in Dulbecco’s Modified Eagle Medium (DMEM) supplemented with 10% fetal bovine serum (FBS) and 1% penicillin–streptomycin. The cultures were incubated under a humidified CO_2_ incubator at 37 °C and 5% of CO_2_.

##### Cytotoxicity Assessment by MTT Assay

To assess the cytotoxic effect of the CECDs, 5000 3T3-L1 mouse embryonic fibroblast cells/well were plated in 96-well plates and allowed to grow until a confluency of about 70%. Cells were exposed to the different concentrations of CECDs and incubated for 24 h in a CO_2_ incubator. After treatment, 10 µL of MTT solution (5 µg/mL) was added to each well, and the plate was incubated in the dark for 3 h. A total of 100 µL of DMSO was then added to dissolve the purple formazan crystals, and the medium was then carefully removed. A microplate reader was used to measure absorbance at 570 nm, and a percentage was calculated to determine cell viability [14].

##### Morphological Observation

For morphological analysis, 1 × 10^6^ cells (3T3-L1) were cultured in 6-well plates and allowed to reach 70% confluency. The 3T3-L1 cells were then incubated with the CECDs for 24 h. After treatment, cells were observed under an inverted light microscope (Euromex, Arnhem, The Netherlands) at 20× magnification.

##### Apoptosis Detection by AO/EtBr Dual Staining

The 3T3-L1 cells that had been treated with the CECDs were stained with a 1:1 solution of Acridine Orange (AO) and Ethidium Bromide (EtBr) (4 μL) to assess apoptosis. After incubating the cells in the dark for over 20 min, they were gently washed with 1× PBS to remove excess dye. The stained cells were subsequently viewed using a fluorescence microscope (Axiovert 5, Zeiss, Jena, Germany).

##### Wound-Healing Assay

3T3-L1 cells were grown in 6-well plates at a density of 1 × 10^5^ cells per well and allowed to reach about 95% confluency. Subsequently, to duplicate a wound, a uniform scratch was made in the center of the well with the sterile 200 μL pipette tip. Carefully aspirated culture liquid was rinsed with phosphate-buffered saline (PBS), after which unattached cells were removed from the wells. Then, 1.5 mL of serum-free media with CECDs or without treatment was added to each well as a control. At the first time point (t_0_) and 24 h later (t_24_), the pictures of the wound area were captured under a phase-contrast microscope with a 10× magnification. Wound closure was analyzed by measuring the distance between wound edges at t_0_ and t_24_ using ImageJ 1.46v software. The effect of CECDs on cell migration was then determined by measuring the percentage of wound closure [15].

##### Statistical Statement

The data have been presented as mean ± standard deviation of the three independent experiments. Statistical significance was analyzed in GraphPad Prism using one-way ANOVA, followed by Tukey’s test.

## 3. Result

### 3.1. Morphological Assessment of Synthesized CECDs

**Figure 2:**
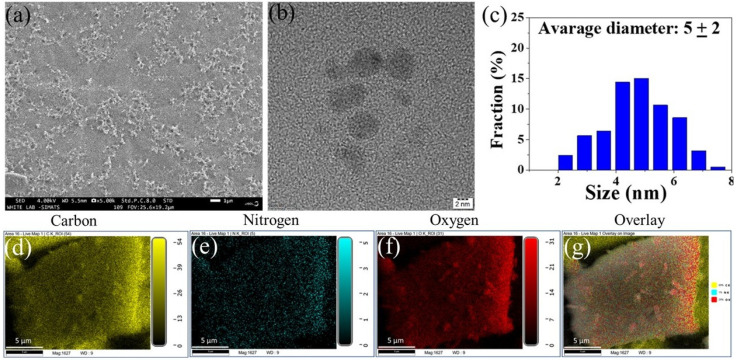
(**a**) FESEM image, (**b**) HR-TEM image, (**c**) particle size distribution histogram of the synthesized CTCDs, (**d**–**g**) display FESEM images from the area of mapping and the associated elemental maps of (**d**) carbon elements, (**e**) nitrogen, (**f**) oxygen and (**g**) the elemental distribution of CECDs overlay.

The morphology, size distribution, and elemental composition of the synthesized CECDs were explained with the help of SEM and TEM. The SEM micrograph (Figure 2a) shows that the surface morphology is rather homogenous and that the nanostructures are distributed evenly and almost spherical, which reflects the lack of agglomeration and that the chitosan matrix gives the necessary stability to it. This type of homogeneous dispersion is beneficial for further application in biology and in surface-interaction. Figure 2b (TEM analysis) can give a more detailed picture of the nanostructure; it shows that almost spherical carbon dots have clear boundaries. The resultant histogram of particle size distribution (Figure 2c) indicates that the size distribution is very limited with the mean diameter of about 5 ± 2 nm indicating that the ultrasmall carbon dots have been successfully synthesized. This is the nanoscale dimension that is especially pertinent to synergized cellular interaction, biofilm penetration and an optimum optical property. Further proof on compositional purity of the CECDs is provided by EDS elemental mapping (Figure 2d–g). The maps of the spatial distribution of carbon, oxygen, and nitrogen show that the spread of these elements in the nanostructure is even and homogeneous. The uniform co-localization in the overlay image represents a good incorporation of heteroatoms at the carbon skeleton probably as a result of the chitosan backbone and eugenol moieties. This kind of heteroatom doping is reported to vary the surface functionality, hydrophilicity as well as the electronic features, and thus increase the physicochemical and potential bioactive performance of the CECDs. All in all, SEM, TEM, and EDX findings overall prove the successful preparation of uniformly sized, heteroatom-containing carbon dots with structurally and compositionally advantageous characteristics.

### 3.2. Structural Characterization of Synthesized CECDs

**Figure 3:**
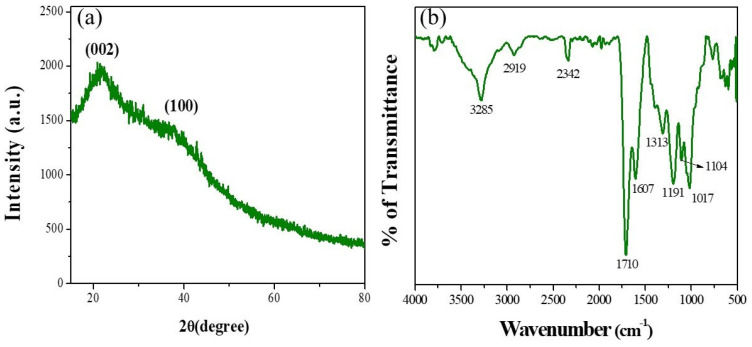
(**a**) FTIR spectrum and (**b**) XRD analysis of CECDs.

It was determined that the structural and chemical properties of the prepared CECDs were clarified by the FTIR spectroscopy and X-ray diffraction analysis (Figure 3). The FTIR spectrum of CECDs (Figure 3a) has a number of some typical absorption bands, in which the existence of a large number of surface functional groups based on chitosan and eugenol precursors is confirmed. The wide band at the middle of the spectral range of the band of 3285 cm^−1^ represents the overlapping O-H and N-H vibrations, which means that the surface of the CECD has hydroxyl and amine functional groups. These polar groups are essential toward increased aqueous dispersibility and surface reactivity. The peak of 2919 cm^−1^ stretching elements is a result of the aliphatic C-H vibrations and the band at 2342 cm^−1^ is related to the adsorbed CO_2_ which is usually present in carbon-based nanomaterials. An intense and sharp peak at 1710 cm^−1^ is related to C=O stretching vibrations, proving the existence of carboxylic acid and/or carbonyl groups. The band at 1607 cm^−1^ is attributed to C=C stretching in aromatic realms which is a manifestation of the partial graphitization of the carbon core. Other peaks at 1313 cm^−1^ and 1191 cm^−1^ are C-N and C-O stretching vibrations, respectively, which are evidence that the nitrogen- and oxygen-containing functionalities were successfully incorporated. C-O-C stretching modes of the bands at 1104 cm^−1^ and 1017 cm^−1^ prove the existence of ether and polysaccharide-derived linkages. All these functional groups confer the CECDs with high hydrophilicity, chemical stability and possible bioactive interactions [16,17].

Figure 3b of the XRD pattern of CECDs shows two broad diffraction peaks at about 2θ ≈ 22–25° and 42–44° corresponding to the (002) and (100) planes of graphitic carbon, respectively. These generally amorphous carbon structures with short-range graphitic organization are indicated by the broadness of these peaks, and this is typical of carbon dots prepared by bottom-up methods. The (002) reflection indicates the cases of disordered graphitic layers, the (100) plane indicates in-plane structural organization of sp^2^-hybridized domains of carbon. This partially graphitized system, which is accompanied by a significant number of surface functionalities, is also conducive to the alignment of optical properties and the interaction with biological systems, and, hence, the appropriateness of CECDs to be deployed against biomedical and antimicrobial uses [18].

### 3.3. Optical Characterization of Synthesized CECDs

**Figure 4:**
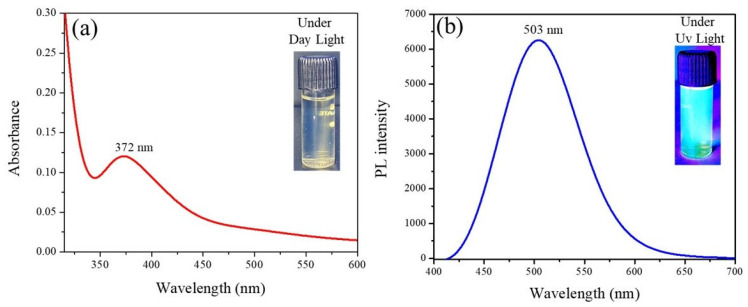
(**a**) UV spectroscopy analysis and (**b**) photoluminescence of CECDs.

UV-Vis absorption and photoluminescence (PL) spectroscopy were comprehensively used to systematically study the optical properties of the synthesized CECDs (Figure 4). Figure 4a demonstrated that the UV-Vis absorption spectrum of CECDs has a strong absorption band at a wavelength of approximately 372 nm, which can be explained by the π-π* transitions of aromatic sp^2^ carbon domains and potentially n-π* transitions of surface carbonyl and heteroatom-containing functional groups. The existence of this absorption property is an indication that conjugated carbon structures and surface states are formed out of the chitosan and eugenol precursors [19].

The photoluminescence properties of CECDs (Figure 4b) indicate that CECDs exhibit strong fluorescence emission when excited by the UV light, with the highest emission point falling at around 503 nm which is bright blue–green fluorescence. The strong PL emission is a possible indication of efficient fluorescent charge carriers that is usually related to surface defect states, heteroatom doping, and quantum confinement effects in carbon dots. The attached inset photos also validate the optical response, which are recognized to be clear and bright under the daylight and fluorescent under the UV light. The high and stable photoluminescence, of CECDs, indicates their possible suitability in fluorescence-based applications such as bioimaging, sensing and microbial labeling, as well as their multifunctional application in antimicrobial and diagnostic systems [20].

### 3.4. Antibacterial Activity of CECDs Against Oral Pathogen (Streptococcus mutans)

**Figure 5:**
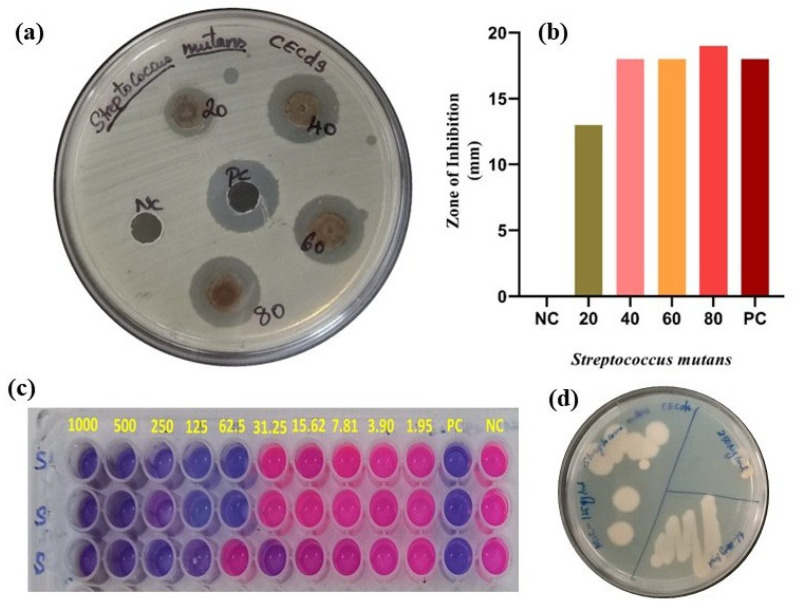
Antimicrobial activity of CECDs. (**a**) Zone of inhibition of CECDs. (**b**) Graph represented for zone of inhibition. (**c**) MIC. (**d**) MBC of CECDs against *S. mutans*.

The antimicrobial effect of the produced CECDs against *S. mutans*, an oral pathogen, was rigorously tested by agar diffusion, MIC, MBC, and viability tests, as illustrated in Figure 5. As seen in the agar well diffusion agar (Figure 5a), the zone of inhibition (ZOI) surrounding the wells with the CECDs was clear and well-defined, indicating their antibacterial properties. It is worth noting that there was progressive enhancement in the inhibition zones with the corresponding increase in the CECD concentration that indicated a strong dose-dependent antibacterial property. Quantitative analysis (Figure 5b) revealed that ZOI values of about 13, 18, 18 and 19 mm were observed with CECDs with the concentrations 20, 40, 60 and 80 µg/mL respectively, though the positive control had a ZOI of 18 mm. Notably, the CECDs at the concentration of both 80 and 100 µg/mL showed a slightly higher inhibition zone than the standard control, indicating its high antibacterial potential with *S. mutans*. These results were further supported by the MIC and MBC assays which revealed better antibacterial performance of CECDs. The obtained values of MIC and MBC were 125 µg/mL and 250 µg/mL, respectively (as revealed in Figure 5c,d), and it was shown that the CECDs have potential to inhibit bacterial growth and induce bactericidal activity at comparatively low concentrations. These results highlight the effectiveness of CECDs as nanomaterials with antimicrobial purposes [21].

Also, SYTO9/Propidium iodide (SYTO9/PI) dual lingering was used to estimate the viability of bacteria to clarify the apprehending action. Bacterial cell death was confirmed by showing that predominantly the cells treated by CECD (PI-positive) demonstrated red fluorescence and this is a sign of cell membrane rupture and cellular damage. Conversely, untreated control cells exhibited almost only green fluorescence (SYTO9-positive), which is a typical attribute of active cells in good condition with fully functional membranes. This positive PI signal of treated samples indicates that the dominant mechanism of the antibacterial activity of CECDs is the disruption of membranes and possibly with the help of functional groups on the surfaces and heteroatom-impregnated carbon frameworks. Taken together, these findings indicate that CECDs have strong antibacterial properties against *S. mutans*, and their use can be considered in oral healthcare and as antibacterial oral products, in antibacterial biofilm approaches, and as a component of antimicrobial dental solution [22].

### 3.5. Bifunctional CECDs for Fluorescence-Guided Antimicrobial Applications

**Figure 6:**
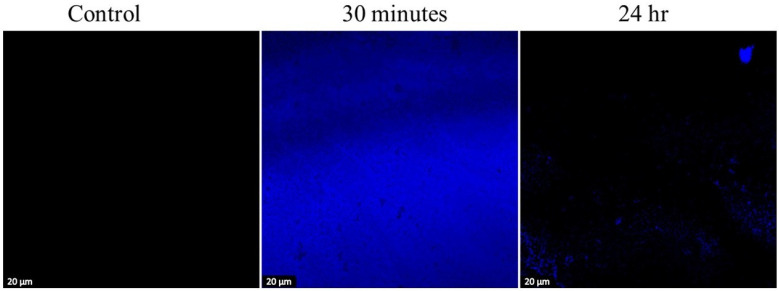
Confocal fluorescence images depicting biofilms with live bacterial cells stained using CECDs as a staining dye. The images show the presence of treatment with 100 μg/mL of CECDs. Scale bar represents 50 μm.

Figure 6 depicts confocal fluorescence microscopy data that show the behavior between CECDs and *S. mutans* biofilms. With CECD treatment, the biofilm has thick and homogenous blue fluorescence, which is a sign of a well-formed biofilm containing living as well as non-living bacterial cells and an intact extracellular polymeric matrix. The standard fluorescence intensity indicates that there is strong cell viability and biofilm integrity throughout the field of observation. Biofilms exposed to CECDs 100 µg/mL on the other hand reveal a significant decrease in the intensity of fluorescence and distortion of the biofilm structure. There is a sparse and uneven fluorescence signal with the treated samples, indicating a poor and broken cellular viability and biofilm matrix. Such a sharp decrease in the density of biofilm means that CECDs will enter the biofilm framework and disrupt the existence of bacteria successfully. The dual characteristics of the latter CECDs as both fluorescent probe and antibiofilm agents are indicative of their dual usage, whereby both biofilm destruction can be directly visualized and the antimicrobial action taken to occur against the microbe. These results suggest the possible use of CECDs in biofilm imaging, real-time fluoroscopy, and therapeutic interventions for controlling oral biofilm-related infection [23,24].

**Figure 7:**
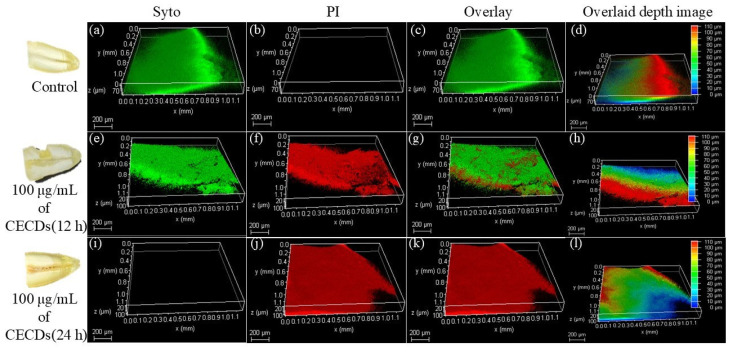
CLSM images showing *S. mutans* biofilm on the tooth surface under control conditions and after CECD treatment (100 µg mL^−1^, 12 and 24 h), with live (SYTO, green) and dead (PI, red) cells. Overlay and depth images reveal CECD-induced biofilm disruption, reduced thickness, and increased bacterial cell death. (**a**) Control–SYTO: dense viable biofilm; (**b**) Control–PI: minimal dead cells; (**c**) Control–Overlay: predominantly live cells; (**d**) Control–Depth: thick uniform layer; (**e**) 12 h–SYTO: reduced viability; (**f**) 12 h–PI: increased cell death; (**g**) 12 h–Overlay: partial disruption; (**h**) 12 h–Depth: decreased biomass; (**i**) 24 h–SYTO: minimal live cells; (**j**) 24 h–PI: dominant dead cells; (**k**) 24 h–Overlay: extensive killing; (**l**) 24 h–Depth: marked thinning and biofilm loss.

Figure 7 shows a 3D CLSM projection of *S. mutans* biofilms established on the tooth surface in the control condition and after different treatments with CECDs 100 µg/mL every 12 and 24 h. A well-structured and densified biofilm structure is observed in the control samples, but with an intense green fluorescence (SYTO staining), which is an indication that the vast majority of the bacterial cells are alive and that the biofilm layer forms a continuous layer, pinacolone, which strongly adheres to the tooth surface. The depth and overlay images further prove that the thickness of the biofilm and the even distribution of the living cells between the enamel surface were significant. By contrast, in CECD-treated samples, there will be a strong modification of biofilm structure. Following 12 h of treatment, there is a distinct loss in the intensity of green fluorescence with an increase in the intensity of red fluorescence (PI staining), which is an indication of the impaired membrane integrity and the advancement of bacterial cell death. The depth profiles and 3D-reconstructions demonstrate the partial disruption of the biofilm, reduction in biomass and distorted surface structure. The effect of 24 h exposure is more dramatic, with the antibiofilm effect being widespread with red fluorescence throughout, few green spots, and a significantly thinned layer of biofilm. The thickness and coverage of biofilm is evidently reduced in overlay images and depth-coded images, showing that CECDs have come into contact with the entire biofilm data. All of these CLSM observations demonstrably and clearly show that CECDs are capable of interfering with *S. mutans* biofilm formation in a time-dependent fashion that triggers bacterial cell death and destabilizes the biofilm structure on the tooth surface. This is due to the potential of CECDs in the future as a multifunctional antibiofilm agent in dental and oral health practice because of their capability to thin the biofilm and their ability to destroy the biofilm [25].

**Figure 8:**
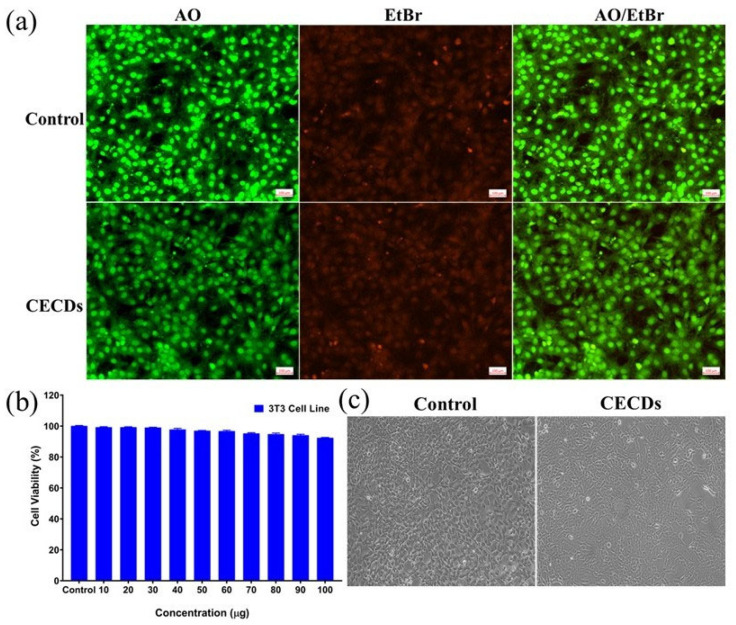
(**a**) Dual staining (AO/EtBr) for live and death cells in 3T3-L1 cell line with and without CECDs. Scale bar at 100 µm. (**b**) Cytotoxicity effect of CECDs in 3T3-L1 cell line by MTT assay. The data were represented by mean and standard deviation of a triplicate experiment. No significant change was observed in the control and CECD-treated groups. (**c**) Morphological changes in CECDs.

Figure 8 is a summary of the in vitro cytocompatibility and cellular response of CECDs with murine fibroblast (3T3-L1) cell line by dual fluorescent staining, metabolic activity analysis, and morphological analysis. The membrane integrity distinction between live and dead cells is clearly observed in the AO/EtBr dual-staining assay (Figure 8a). In the control, it was observed that the cells mostly had bright green fluorescence (AO-positive), which showed a large proportion of viable cells with intact membranes and a healthy nucleus, whereas red fluorescence of EtBr was insignificant. On the same note, the green fluorescence was highly predominant in the CECD-treated cells at the higher concentrations, thus confirming the fact that there is no significant membrane damage or apoptosis when exposed to CECD. This represents a good sign that shows that the cytocompatibility of the nanomaterial is good and that there is minimal cell death in the treated samples because of the absence of substantial red fluorescence [26].

The cytocompatibility of the synthesized CECDs was systematically measured by using the MTT metabolic activity assay to measure the possible dose-dependent effects on 3T3-L1 fibroblasts (Figure 8b). Across all the concentrations tested to 100 µg/mL, cell viability was consistently greater than 90% and consequently displays a very favorable biocompatibility profile with only small reductions in viability which are not of clinical relevance at the highest levels of exposure. These findings show that CECDs have no adverse effects on mitochondrial metabolic function and are well-tolerated by mammalian fibroblasts at the investigated concentration range. Phase-contrast analysis by microscopy confirmed these further and showed that both control and CECD-treated cells retained their characteristic fibroblast morphology in terms of their elongated spindle-shaped structures and normal surface adherence and cellular spreading (Figure 8c). Even at 100 µg/mL, only minor morphological alterations such as slight cell rounding in a small fraction of the population were observed, suggesting mild and reversible cellular stress, not cytotoxicity. Importantly, no evidence of cell shrinkage, membrane disruption, dissociation or loss of confluency was detected in any adaptational treatment group. There is no significant difference in the antibacterial or cytocompatibility effects of CECDs and control materials. Collectively, these observations confirm that CECDs have excellent compatibility with fibroblast cells, in which they maintain the integrity of the plasma membrane, viability, and normal cellular architecture even at quite high concentrations. This corpulent safety profile coupled with their displayed antimicrobe and antibiofilm performance support the potential usefulness of CECDs as biocompatible nanomaterials for future leverage in dental therapeutics and wound management as well as infection control strategies.

**Figure 9:**
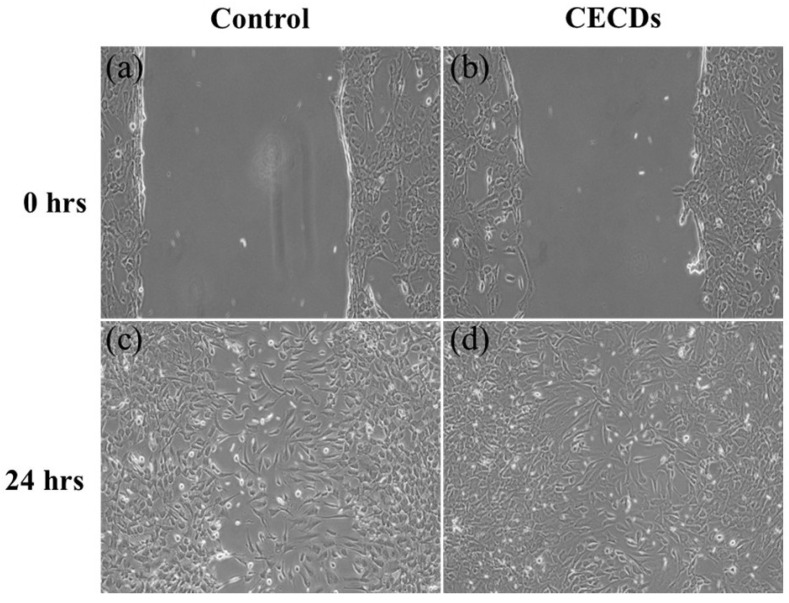
In vitro scratch (wound healing) assay demonstrating migration of 3T3-L1 fibroblast cells under (**a**,**c**) control, and (**b**,**d**) CECD-treated group, showing accelerated wound closure after 24 h without compromising normal cell morphology.

Figure 9 illustrates the effect of CECDs on fibroblast migration using an in vitro scratch (wound healing) assay in 3T3-L1 cells. At 0 h, both control and CECD-treated groups exhibit a clearly defined scratch area with comparable wound widths, confirming uniform initial conditions (Figure 9a,b). The cells at the wound edges display typical fibroblast morphology, indicating that the scratching process did not induce excessive cellular damage. The difference in wound closure is significantly different between the groups after 24 h of incubation (Figure 9c,d). The negative cells display partial cell migration into the scratched area, and the gap is closed but not completely. Conversely, the cells treated with CECD have an increased migratory behavior with a significantly reduced wound area and the cells populate the area with a greater density. The cured cells maintain their elongated and spindle-shaped fibroblast morphology implying active movement and proliferation and not morphological alterations related to stress. These findings suggest that CECDs have no effect on the motility of fibroblasts and, on the contrary, they seem to have a small stimulatory effect on cell movement and wound repair. The improved wound-healing process can be explained by the positive surface chemistry and biocompatibility of CECDs that promotes cell adhesion, spreading, and reorganization of the cell cytoskeleton. On the whole, the wound-healing test proves that CECDs exhibit cytocompatibility and potentially can positively affect fibroblast migration, which supports their possible use in biomedical and dental practice, where tissue repair and regeneration are required [27,28].

## 4. Discussion

The present research establishes CECDs as a rationally well-designed multifunctional nanoplatform that combines favorable physicochemical features with biologically relevant activity solving two important problems in current dentistry, including the ability to control infection and to promote tissue repair [29]. Detailed morphological and compositional analyses verified the successful synthesis of heteroatom-doped carbon dots with good elemental homosensitization distribution, spherical morphology, and ultrasmall size ca. 5 ± 2 nm proved by SEM, TEM and EDX characterization. Such nanoscale dimensions, coupled with a high surface area to volume ratio, are well known to be essential factors in increased interaction with microbial biofilms and in mammalian cells [30]. The introduction of nitrogen and oxygen functionalities derived from the chitosan and eugenol precursors were responsible for the enhanced hydrophilicity, colloidal stability and surface reactivity as well as modulating the electronic structure of the carbon core. These features represent the basic characteristics for effective biofilm penetration and cellular internalization, and for electrostatic interactions with the negatively charged bacterial membranes, which are in agreement with some previously reported behaviors of heteroatom-doped carbon dots for biomedical systems [31].

The multifunctional nature of CECDs was further confirmed from the structural and optical characterization. FTIR and XRD analyses showed the presence of a partially graphitized amorphous carbon skeleton decorated with hydroxyl, amine, carbonyl and ether functional groups that endows molecular chemical stability, aqueous dispersibility and inherent biocompatibility [32]. Such surface functionalities are known to aid in conjugation with biological targets (as well as such interfacial interactions in contact with complex biological environments). Optical studies revealed characteristic movements of π-π* transitions in the UV-Vis region and a strong emission of a luminous (blue–green) migration band, which is typical for the existence on the surface of well-passivated surface states and quantum confinement processes. These optical properties are extremely beneficial for fluorescence-guided antimicrobial applications and bioimaging as they can form a simultaneous visualization and therapeutic intervention [33,34]. Collectively, the obtained physicochemical and photophysical properties are very similar to previous publications on bio-derived carbon dots, and prove the suitability of CECDs as a versatile platform integrating diagnostic and therapeutic functions in the oral cavity.

The stable and intense photoluminescence shown by the CECDs allows their simultaneous use as therapeutic agents and imaging probes and allows the real-time visualization of microbial biofilms coupled with antimicrobial action. Such an intrinsic fluorescence-guided functionality is particularly useful in dental applications, in which diagnostic monitoring and therapeutic intervention are often to be performed in the spatially restricted and biologically complex oral environment. This obtained antibacterial and antibiofilm activity depicted in the current study is associated with the cooperative contribution of the two ligands incorporated through the CECDs. CECDs were shown to have marked, concentration-dependent inhibitory effects on *S. mutans*, including low MIC and low WBC values for inhibition and killing of the bacteria, respectively, which confirmed the effective suppressive bacteria growth and bactericidal effect at relatively low dosages. These results are similar to the previous reports of carbon dot-based nanomaterials with strong activity against oral pathogens [35,36,37,38].

Furthermore, confocal and CLSM analyses provided direct visual evidence of progressive biofilm disruption, reduction in the thickness of biofilm structures, and increase in bacterial cell death following exposition of CECD that agrees with their capacity to penetrate and destabilize mature biofilms. The antimicrobial activity of CECDs is likely to be mediated by several, inter-related modes (electrostatic interactions between positively charged surface functionalities and negatively charged bacterial membranes, increased membrane permeability and induction of oxidative stress involving reactive oxygen species (ROS) formation associated with the CECDs derived moieties). This multimodal mechanism of action decreases the likelihood of developing resistance, and demonstrates the benefits of CD-based strategies compared to routine antibiotics affording to handle biofilm-associated infections of the oral cavity [39,40].

Equally important, CECDs showed excellent cytocompatibility towards mammalian fibroblasts suggesting their biomedical use. In vitro tests showed that the viability of 3T3-L1 fibroblasts remained more than 90% even when they were treated at concentrations as high as 75–100 µg/mL, without membrane damage and without abnormal change in the normal spindle shape of the cell morphology. Moreover, CECD-treated cells showed augmented migratory behavior in scratch assays and thus play a potentially conducive role in closure and cellular repairing carrier processes [41]. The combination of the antimicrobial, antioxidant, and bioactive surface nature of CECDs is likely to lead to a microenvironment that is favorable for cellular proliferation, extracellular matrix remodeling and tissue regeneration. Taken together, these findings make CECDs promising candidates for the next generation of dental nanotherapeutics with the ability to control infection and promote tissue repair simultaneously to meet a critical unmet need in modern oral healthcare.

## 5. Conclusions

This study successfully developed and validated chitosan–eugenol-derived carbon dots (CECDs) as a multifunctional nanoplatform with significant relevance to contemporary dental therapeutics. The synthesized CECDs exhibited uniform spherical morphology, ultrasmall dimensions (~5 ± 2 nm), stable photoluminescence, and effective heteroatom-doped surface functionality, as confirmed through comprehensive physicochemical characterization. Functionally, CECDs demonstrated potent antibacterial and antibiofilm activity against *S. mutans*, achieving meaningful microbial inhibition at relatively low concentrations, with MIC and MBC values of 125 µg/mL and 250 µg/mL, respectively. Confocal imaging further verified their capacity to penetrate and disrupt mature biofilms, highlighting their potential for fluorescence-guided antimicrobial interventions. Importantly, CECDs maintained excellent cytocompatibility, preserving fibroblast viability, normal cellular morphology, and membrane integrity across all tested concentrations, while also promoting enhanced cell migration in wound-healing assays. These findings collectively indicate that CECDs uniquely combine antimicrobial efficacy with favorable biological safety, addressing a critical clinical need for materials capable of both infection control and tissue-supportive functions. Compared with conventional antimicrobial strategies, the integrated carbon dot system offers the advantages of stability, biocompatibility, and multifunctionality, underscoring its translational promise for oral healthcare applications. Future investigations focusing on detailed mechanistic validation, long-term biosafety assessment, and in vivo performance are warranted to further advance CECDs toward clinical implementation in preventive and regenerative dentistry.

## Data Availability

The original contributions presented in this study are included in the article. Further inquiries can be directed to the corresponding author.
